# Personality Traits and Escape Behavior in Traffic Accidents: Experiment and Modeling Analysis

**DOI:** 10.3389/fpsyg.2021.800093

**Published:** 2022-01-31

**Authors:** Yaohua Xie, Xueming Xu, Wenjuan An

**Affiliations:** ^1^National Engineering and Research Center for Mountainous Highways, Chongqing, China; ^2^China Merchants Chongqing Communications Technology Research & Design Institute Co., Ltd., Chongqing, China; ^3^School of Electronic and Information Engineering, Southwest University, Chongqing, China; ^4^School of Civil Engineering, Chongqing Jiaotong University, Chongqing, China

**Keywords:** personality traits, traffic accidents, modeling, individual escape behavior, behavior analysis

## Abstract

In this article, we tried to reveal the relationship between personality traits and escape behavior in traffic accidents. Different from common computer simulations, this study, for the first time, established a real database recording the escape behavior and personality traits of subjects when watching a first-person-view driving video with explosion. Then, we used a modeling method of general linear to establish a quantitative model of the influence of personality traits, explosion, and their interaction on escape behavior. In the model, we introduced escape response time, escape time, escape direction, escape speed, escape trajectory, and other motion characteristics to study individual escape behavior in accidents. Through the analysis, we concluded several conclusions, including that high neurotic individuals tend to escape with shorter response time and slower speed by choosing doors far from the explosion source. These conclusions may provide some references for the effective escape of the crowd and the successful escape of the individual under traffic accidents.

## Introduction

The research on the escape behavior of people in traffic accidents is a hot topic in the development of transportation. With the rapid growth of economy, the transportation industry, which is convenient for people to travel, has also been explosively developed. At the same time, the frequency of extreme traffic accidents is also getting higher and higher, such as multivehicle collision on the fast road, automobile explosion in the tunnel, or tire explosion accidents. In extreme and traffic accidents, the escape ability of an individual is very important. The effective, rapid, and safe escape of individuals has become indispensable basic theoretical research for the sustainable development of traffic. The successful escape of individuals and the successful escape of crowds under extreme events are inseparable from the revelation of the escape behavior rules of people in traffic accidents.

In fact, behavior of humans in a disastrous situation has been studied after the fact and has been documented by sociologists and a psychologist (Keating, 1982; Vaught and Wiehagen, 1991; Elliott and Smith, 1993). Because the escape behavior of people is susceptible to different personality traits of individuals, it is of great significance to study the mapping relationship between individuals with different personality traits and escape behavior in the field of security. Through our investigation, it is found that there is no report on the relationship between individual personality traits (the Big Five) and escape behavior today. In addition, the research on escape behavior in accidents is mostly based on computer simulation, which has limited guiding significance for the analysis of escape behavior in actual accidents. Therefore, to study the influence of extreme scenes on psychological-escape ability, we induced an emotional shock in the subjects by letting them watch the first-view video of a driver containing traffic accident in a close distance in the laboratory environment and observed their escape behavior. This article aims to reveal the law of individual escape behavior affected by different personality traits and explosion scenarios through controllable experiments and mathematical models.

Our contributions are as follows:

1. Different from common computer simulation, this study adopts the experiment of designing extreme traffic accidents without or with explosion scenes to establish a real database of the escape behavior and individual personality traits of 200 subjects.
2. Most of the existing studies on escape behavior only analyze one or two of the motion characteristics, such as response time, escape time, escape direction, escape speed, and escape trajectory, while ignoring the integrity analysis of the motion characteristics of escape behavior. In our study, we introduced escape response time, escape time, escape direction, escape speed, escape trajectory, and other motion characteristics to study the individual escape behavior in traffic accidents.
3. As the first related study, to obtain the mapping relationship between individuals with different traits and escape behavior, we adopted the modeling method of general linear model and established a quantitative model of the influence of personality traits, explosion, and their interaction on escape behavior. We found that high neurotic individuals tend to escape with a shorter response time and slower speed by choosing doors far from the explosion source. This will provide some reference for the effective escape of the crowd and the successful escape of the individual under traffic accidents.

## Related Research

Every year, a large number of casualties and property losses are caused by traffic accidents, fires, explosions, and other emergencies worldwide, and the processing cost of these emergencies is very huge. Escape behavior in unexpected situations has always been a problem that researchers pay more attention to. Emergency escape involves moving a large number of people in response to natural disasters (such as fires, floods, or terrorist attacks). If not planned, escape may cause congestion at the exit. At the same time, to minimize casualties and property losses (Koo et al., 2013), it is necessary to adopt appropriate and efficient escape schemes of people and vehicles. Generally, the research of crowd evacuation mainly includes real experiments and model-based simulations. Experimental research in bottleneck (Hoogendoorn and Daamen, 2005), classroom (Zhang et al., 2008), and high-rise building scenarios (Ma et al., 2012) has revealed many evacuation behaviors and characteristics under normal situations. The escape model of the library (Cai, 2018) was established by using the evacuation software Pathfinder, and the escape process was simulated to obtain the movement time required for the escape of people at all levels. Through the comparison and analysis of the dangerous critical time obtained by the previous fire simulation, the safety escape of people in each fire scenario was judged. Finally, the corresponding measures were proposed. The different psychological response of different groups in the face of fire, and the different effects on escape were analyzed by Zhang (2016). According to the discrete selection model of crowd evacuation and based on the scale of Xiwang Station, the environmental factors of escape of people were set, and the most unfavorable escapees were selected as the individual model to calculate the time required for their escape. Finally, according to the fire simulation results and the calculation results of personnel escape time, suggestions were put forward for the layout of station fire facilities and the selection of personnel escape path. Li and Wang (2011) studied the safety escape time of trapped people, the influence of high-temperature smoke, the utilization rate of tunnel transverse passage, and the characteristics of escape behavior of people by using the empirical calculation theory and the calculation software Building EXODUS. So far, numerous models of crowd simulation have been proposed. Integrating emotion and personality into the simulation of agents becomes a popular method of crowd simulation (Durupinar et al., 2016). Liu et al. (2009) presented a model for studying the impacts caused by emotions of individual agents in a crowd. In their research, the method of joining a crowd by an individual was proposed, and the reactive mechanism between crowds was also explored. Braun et al. (2003) presented a model for studying the impact of characteristics of individual agents in emergent groups, on the evacuation efficiency as a result of local interactions. Mao et al. (2019) considered the effects of stress on behaviors of individuals based on the emotion contagion model. Pelechano and Badler (2006) gave a leader-based crowd model. Durupinar et al. (2011) introduced a personality-based crowd model. Durupinar et al. (2008) specified behavioral adjectives from each type of personality “OCEAN,” then they made directly the relationship between the system parameters and the personality traits. Qi et al. (2020) studied the human function index in the plateau environment, determined the maximum oxygen uptake as the main factor affecting the human motor function, and determined the escape rate of people in the plateau environment through theoretical derivation. Their results showed that with the increase of altitude, the oxygen uptake of human body gradually decreased, and the escape rate of people decreased with the increase of altitude, which was significantly related to gender and age. Chen and Shao (2015) explored the changes between nonescape behavior and escape behavior, and the relationship between escape behavior and the location of escape events. The method they proposed can estimate the position of the divergence center when the escape occurs. The performance of the proposed method was verified by using multiple datasets. Ali and Shah (2007) studied the random escape time in emergency situations and analyzed the probability distribution of escape time in a simple closed fire scenario. Their results showed that escape time is variable, which are affected by many uncertain factors. The escape process in an emergency is a complex process. Although human behavior is similar under panic conditions, escape time is also affected by the emergency evolution dynamics of a given environment. First, Chu and Wang (2011) used an Unmanned Aerial Vehicle and tracking technology to capture pedestrian flow and extract pedestrian trajectory. Second, a top-down hierarchical clustering strategy was proposed to group people and solve the problem that small groups found difficult to determine. To solve the problem that most existing neural networks use one-dimensional vectors to model and cannot learn the spatial information of pedestrians, Song et al. (2021) proposed tensor to represent the basic environmental characteristics of pedestrians. They design a deep convolution long short-term memory (LSTM) network to predict the spatio-temporal trajectory sequence. Their experimental results showed that the network can estimate the trajectory of escape and countercurrent crowd more realistically.

Sarwar and Jaffry (2017) proposed that the social force model (SFM) has the ability to simulate the movement of pedestrians in the normal situation of panic. At the same time, they proposed that there is almost no figure of personality traits in the previous pedestrian model research on crowd simulation, but they believed that the personality traits of pedestrians are also a key factor, and they should be modeled to realistically simulate the movement of pedestrians. They carried out experiments and found that if the personality traits of pedestrians in the crowd are different, the escape behavior will become complex and chaotic. In the previous literature, the best escape time of SFM is obtained when the overall panic value is 0.4, but after combining the neuroticism of pedestrians and conducting detailed experiments on homogeneous and heterogeneous people, the best escape time is obtained when the overall panic value is 0.3. Their research shows that the neurotic dimension of personality traits has an impact on the escape behavior of a pedestrian. Inspired by their work, we introduced the Big Five Personality Inventory, State Anxiety Inventory, Trait Anxiety Inventory, and Beck Depression Inventory in our study. The results of the inventory are involved in the analysis of individual escape behavior.

In summary, the existing research on escape behavior mainly focuses on the study of group escape behavior in traffic accidents and rarely studies individual escape behavior, especially on the analysis of individual escape behavior with different personal traits. Therefore, this article focuses on the escape behavior of individuals with different personal traits in traffic accidents.

## Construction of Database

In this study, a 2 × 2 factor experiment with low or high personality dimensions and with or without explosion scenarios is used to construct a database. The independent variable is personality traits (i.e., Big Five Personality), and the dependent variable is escape behavior (i.e., escape response time, speed, and direction). Experimental subjects and experimental scenarios are discussed below:

### Database Participants

A total of 200 healthy college students were included in the analysis of this study, including 68 males and 132 females, with an average age of 23.1 years, standard deviation of 3.03, and age range of 18–34 years. All subjects had normal visual acuity or corrected visual acuity, normal hearing, normal color vision, right-handedness, no state-trait anxiety, no previous and current manic episodes, no history of alcohol, drug abuse, developmental delay, and other organic diseases. This study was approved by the Ethics Committee of Southwest University. Before the formal experiment, all subjects signed informed consent.

### Acquisition Scene

**FIGURE 1:**
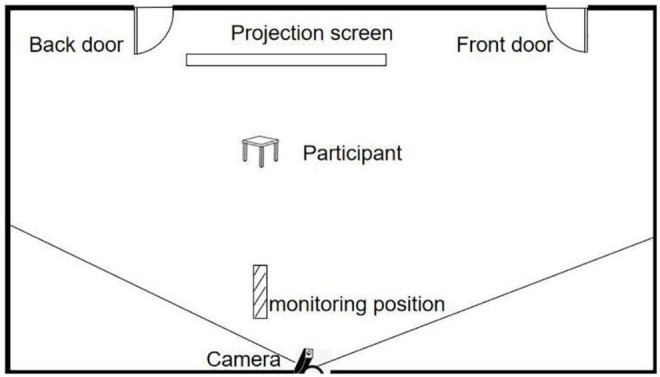
The plane diagram of the experimental scene.

The plane diagram of the experimental scene in this study is shown in Figure 1. First, a more spacious laboratory was selected. Second, video materials were placed in the middle of the laboratory to play TV (78-inch color large-screen TV, screen resolution: 3,840 × 2,160, 2 m away from the subject). There was a high-fidelity sound playing explosive audio below the TV (Soaiy SA-T19 bass Bluetooth sound). There are two exits on both sides of TV and audio, namely, the back door and the front door. The back door is close to the explosion source, and the front door is far from the explosion source, which is used to simulate the choice of individual escape direction after the occurrence of traffic accidents. Finally, the participants were sitting on the screen, watching videos and audio stimuli. The escape behavior of the participants was recorded using the camera (Spedal with high-definition wide-angle camera) behind them, and the escape response time, escape speed, and escape direction were obtained.

### Acquisition Process

All participants first participated in the Big Five Personality Inventory, State Anxiety Inventory, Trait Anxiety Inventory, and Beck Depression Inventory. First, according to the score, subjects without state anxiety, trait anxiety, and depression participated in the experiment. Second, the experimenter would not tell them the process of the experiment and asked the subjects to sign informed consent voluntarily. Finally, the subjects were told to watch a video of the traffic driving scene. When the subjects sat on a stool 2 m away from the screen to watch the driving video, the subjects watched the video and recorded the whole behavior process of the subjects through the high-definition wide-angle camera (if there was no spark in the video material, please escape from the laboratory. If there was spark in the video material, please run away from the laboratory by yourself). To simulate the real traffic accidents, two experimental videos were used as experimental stimulus materials. The two videos were real road driving videos and had a good ecological validity. Among them, the first video was a 2 min self-driving tour video, and the whole process was silent. The second video material of 2 min was homogeneous with the first video. The only difference was that it inserted a 4 s explosion video and audio in 1 min 56 s. The two video materials were played in the experiment in a reverse balanced order to balance the confusion caused by the playback sequence of video materials.

## Database Processing

### Personality Traits Data

According to the completed Big Five Personality Inventory, the personality traits data of all participants were recorded in the database.

### Video Processing of Behavior

The research of human activity recognition through video attracts increasing attention (Liu et al., 2018; Wang K. X. et al., 2020). In the course of the experiment, as the camera collected a complete video of the experimental process, all the behavior of the participants was recorded. To intercept the behavior of the participants leaving the scene from a large number of original videos, we adopted the multiobjective tracking (MOT) system proposed by Wang Z. L. et al. (2020). The tracking system was built based on Jointly learning the Detector and Embedding model (JDE). The traditional MTO methods consist of a detection model and appearance embedding model for data association. The JDE proposed by Wang Z. L. et al. (2020) can learn the target detection and appearance embedding in one model. The model can output both detection results and corresponding embedding results at the same time. An association method was also proposed by Wang Z. L. et al. (2020) to aid the JDE. The architecture of feature pyramid network (Lin et al., 2017) was used as the backbone of JDE. Feature maps at three scales (i.e., 1/32, 1/16, and 1/8 down sampling rate) were obtained from an input video frame to the JDE. Then, the feature maps were used. And finally, the prediction heads were added on fused feature maps at all scales.

Through this method, we can capture the moving coordinate information of the participants from starting to leave the scene, i.e., the moving trajectory of individual, which can be used to extract the motion characteristics such as the direction of escape, the response time of escape, the escape time, and the escape speed in the next step. After obtaining the moving trajectory of each participant, the two annotators independently recorded the ID of participants and calibrated the key frames for the trajectory of the nonescape behavior and the escape behavior. For the trajectory of the nonescape behavior, the two coders recorded the frames *f*_1_ when the participants watched in the video in calm, the frames *f*_2_ when the participants got up after watching the experimental video, and the frames *f*_3_ when the participants walked to the safety zone at normal speed. For the escape behavior trajectory, two coders recorded frame *f*_4_ when the explosion image appeared in the experimental video, frame *f*_5_ when the participants made up their posture and the frame *f*_6_ when the participants ran to the safe zone. Each participant corresponds to a set of arrived data from *f*_1_ to *f*_6_. For the arrived data of each participant from *f*_1_ to *f*_6_, if the difference of the frame number marked by two coders was greater than 5 frames, the two coders negotiated to recalibrate until the two coders reach agreement. If the difference between the frame number marked by two coders is less than or equal to 5 frames, the average number marked by two coders is taken as: $f_{n}=\frac{f_{n1}+f_{n2}}{2}\left(n=1,2,3,4,5,6\right)$. Among them, *f*_*n*1_ represents the number of frames labeled by the first coder, *f*_*n*1_ represents the number of frames labeled by the second coder, and *f_n_* represents the average of the serial number labeled by the two coders. If the value of *f_n_* is not an integer, the nearest integer of *f_n_* is taken.

### Data of Escape Behavior

First, the escape direction can be obtained according to the trajectory of the participants. There are two escape directions in the experimental scene. The escape direction 1 corresponds to the door with a distance of 3.3 m, and the escape direction 2 corresponds to the door with a distance of 4.4 m.

Then, according to the statistical key frames in 4.2, we can first calculate the response time of the nonescape and escape.

The nonescape response time is $t_{p}=\frac{f_{2}-f_{1}}{f_{r}}$, and *f_r_* represents the frame rate of the video and *f*_2_ − *f*_1_ represents the frame number difference between the frame number of the completion of the nonexplosion experimental video and the frame number of the participants to make the posture. The data obtained by dividing the frame number difference by the frame rate can represent the time from the completion of the experimental video to the participants to make the posture, that is, the response time of the subjects under normal conditions.

The escape response time is $t_{p2}=\frac{f_{5}-f_{4}}{f_{r}}$, and *f_r_* represents the frame rate of the video and *f*_5_ − *f*_4_ represents the frame number difference between the frame number of explosion image in the experimental video and the frame number of the participants to make the posture. The data obtained by dividing the frame number difference by the frame rate can represent the time from the explosion image in the experimental video to the subjects to make the posture, that is, the response time of the participants to the stimulation of the explosion image.

Similarly, we can calculate the escape time of nonescape and escape behavior. *t_m_* represents the time spent by individuals escaping from the location of a traffic accident to a safe zone. For nonescape behavior: $t_{m}=\frac{f_{3}-f_{2}}{f_{r}}$, and the escape time for escape behavior is $t_{m}=\frac{f_{6}-f_{5}}{f_{r}}$.

Finally, we can calculate the escape speed by the following formula: $\text{v}=\frac{S}{t_{m}}$, *S* and *t_m_* have been given.

### Data of Database

The data in the final database include the Big Five Personality scores (i.e., neuroticism, extraversion, openness, agreeableness, and conscientiousness) of individual participants, the experimental scene data with and without explosion, the emotional impact scores (i.e., valence and arousal) of individuals, the data of individual escape behavior [i.e., escape response time (s), speed (m/s), and direction (near door 0, far door 1, and distance from the explosion source) (Table 1, the experimental data of five sample participants)].

**TABLE 1:** The experimental data of five sample participants.

	Big Five personality scores					Emotion and escape without explosion^1^					Emotion and escape with explosion^1^				
Number	Neuroticism	Extraversion	Openness	Agreeableness	Conscientiousness	Valence	Arousal	Response time	Speed	Direction	Valence	Arousal	Response time	Speed	Direction
1	42	32	60	30	49	4	1	0.47	0.98	0	−2	5	0.63	1.80	0
2	45	25	33	41	35	1	2	1.00	0.83	1	−1	2	0.40	2.49	1
3	30	42	40	45	41	1	1	0.2	0.94	1	−2	5	0.23	1.55	1
4	45	33	43	40	35	−2	2	0.9	0.73	0	−1	3	0.57	1.18	0
5	21	49	40	47	58	4	1	0.3	0.85	0	−3	8	0.37	1.90	0

*^1^Escape response time (s), speed (m/s), and direction (near door 0, far door 1).*

## Modeling

To reveal the law of escape behavior of people with different personality traits in traffic events, the project designed a 2 × 2 factor experimental design (i.e., low or high personality dimension and with or without explosion experimental group), collected the relevant data of 200 samples, and used the modeling method of the general linear model to establish the theoretical model of escape behavior affected by personality, explosion, and interaction. In the view of the regular trend of exploring the individual escape behavior affected by personality and explosion, the general linear model itself has a wide range of application, simple, and difficult to over-fit. On the basis of the experimental data, the optimal mathematical model is established. To apply to the diversity of the value of escape behavior and emotional dependent variable in this project (i.e., the value of escape response time and speed is continuous, and the value of escape direction is discrete), the general linear model of the project divides the dependent variable y into its probability distribution and its expected value to describe the escape behavior model. This article takes the neuroticism dimension of personality as an example to explain the modeling process.

The main effect models of personality neuroticism and experimental scene on escape behavior (i.e., escape response time, speed, and direction) are as follows:


(1) {μ=c1v1*+c2v2*b∼pdf(μ,σ2)

Among them, (v_1_, v_2_) is the grouping vector of personality neuroticism dimension P or explosive experiment E. The first component is 1, and the second component is 0, indicating low neuroticism individuals and vice versa. The first component is 1, and the second component is 0, indicating that there is no explosion scenario and vice versa; c_1_ and c_2_ represent the effect of personality neuroticism dimension P and explosion scene E on dependent variables, respectively. The statistical significance of c_2_−c_1_ represents the main effect of personality traits or explosion experiment conditions. b is the dependent variable, which refers to one of the escape response time, direction, and speed variables in the experiment. When the value of behavioral variable b (i.e., escape response time and escape speed) is continuous, it is generally assumed that the probability distribution of the variable is normal distribution, and μ represents its mean. When the behavior variable b (i.e., escape direction) has two choices, it is generally assumed that the probability distribution of its variable obeys is binomial distribution, and μ denotes the probability of its value. Considering the interaction effect between the emotion and experimental scene, the project extension model 2 is as follows:

### Interaction Effect Model


(2) {μ=PC1*+EC2*+PEC3*b∼pdf(μ,σ2)

PE is the interaction matrix of personality neuroticism and experimental scene. C_1_, C_2_ and C_3_ represent the effect matrix of personality, explosion scene, and their interaction on escape behavior b. Models 1, 2 represent the general linear model (i.e., main effect and interaction effect) of personality neuroticism and experimental scene on escape behavior, which is used to describe the trend between personality traits and experimental influence on escape behavior. To further understand and show the effect of each factor in the model, this article further expands the effect of each item and the corresponding coefficient and obtains the following full variable model with independent variables and their interaction items.

### Full-Variable Models

To explore the influence of personality neuroticism and explosion scene on the escape behavior tendency of an individual, a general linear model is used to establish a full-variable impact model of low or high neuroticism with or without explosion scene and their interaction on the escape behavior (i.e., response time of escape, escape speed, and escape direction) of subjects. The full-variable impact model is as follows:


(3) $$\left\{ \begin{array}{c} \mu \text{=}{\text{c}}_{1}{\text{N}}_{\text{L}}+{\text{c}}_{2}{\text{N}}_{\text{H}}+{\text{c}}_{3}{\text{E}}_{\text{N}}+{\text{c}}_{4}{\text{N}}_{\text{Y}}+{\text{c}}_{13}{\text{N}}_{\text{L}}{\text{E}}_{\text{N}}\\ +{\text{c}}_{14}{\text{N}}_{\text{L}}{\text{E}}_{\text{Y}}+{\text{c}}_{23}{\text{N}}_{\text{H}}{\text{E}}_{\text{N}}+{\text{c}}_{24}{\text{N}}_{\text{H}}{\text{E}}_{\text{Y}}\\ \text{b}∼\text{pdf}\left(\mu ,\sigma^{2}\right) \end{array}\right.$$

Among them, N_L_ and N_H_ are low and high variables of neuroticism of subjects (value 1,0 represents low neuroticism and value 0,1 represents high neuroticism). E_N_andN_Y_ are nonexplosion and explosion scene variables (i.e., value 1,0 means nonexplosion scene and value 0,1 means explosion scene). N∗E∗ represents the interaction between the neuroticism and the test scenario groups. c∗ and c∗∗ are the effects of their variables on individual escape behavior b. b is one of the experimental dependent variables, which refer to the escape behavior (i.e., response time, escape speed, and escape direction). When b takes the response time of escape and escape speed, its probability distribution is assumed to be a positive distribution, and μ represents its mean value. When b takes the escape direction, the probability distribution is assumed to be binomial distribution, and μ denotes the probability of choosing a distant gate far from the explosion source. Based on the experimental data of the project, models 1–3 are taken as the theoretical guidance, and the theory and algorithm of general linear modeling are used to estimate the specific values of model parameters and the high-density interval of parameters. The independent variables that have a significant impact on the escape behavior are retained to obtain the optimal quantitative model.

### Optimal Model

Based on the experimental data, through the general linear model theory and parameter estimation algorithm, the project obtains the model of emotional impact and escape behavior tendency of individuals in neuroticism and explosion scene: low or high neuroticism with explosion scene and high neuroticism and explosion interaction. These four items have significant effects on emotional impact and escape behavior, so the optimal model of the project has the following forms:


(4) $$\left\{ \begin{array}{c} \mu ={\text{c}}_{1}{\text{N}}_{\text{L}}+{\text{c}}_{2}{\text{N}}_{\text{H}}+{\text{c}}_{4}{\text{E}}_{\text{Y}}+{\text{c}}_{24}{\text{N}}_{\text{H}}{\text{E}}_{\text{Y}}\\ \text{b}∼\text{pdf}\left(\mu ,\sigma^{2}\right) \end{array}\right.$$

The other four dimensions (i.e., extroversion, openness, agreeableness, and conscientiousness) of personality traits and experimental explosion scenarios affect the emotional experience and escape behavior of subjects. The project replaces the N representing the neuroticism in the model 4 in turn to represent extroversion Ex, openness O, agreeableness A, and conscientiousness C and then obtains a full-variable model of the interaction between extroversion, openness, agreeableness, conscientiousness, and experimental explosion factors. Through the parameter estimation and variable optimization theory of the general linear model, on the basis of the experimental data, the optimal mathematical model of the influence of personality and explosion on the emotional impact and escape behavior of subjects is finally obtained, and the regular trend of the influence of different personality traits on the emotional impact and escape behavior of subjects is revealed.

## Results

Using the general linear modeling method, based on the experimental data of 200 subjects, this study obtained the mathematical models of personality neuroticism N, extroversion Ex, openness O, agreeableness A, conscientiousness C, and experimental explosion E affecting the escape behavior (i.e., response time T, speed v, and direction D) and emotional impact (i.e., valence V and arousal A), including the main effect model and interaction effect model. The main effect model contains six and the interaction effect model contains five specific models.

### Results of Modeling

Personality neuroticism N, extroversion Ex, openness O, agreeableness A, conscientiousness C, and experimental explosion E affect the escape behavior (T, v, D) of subjects and emotional impact (V, A) of the significant main effect mathematical models are as follows: Tables 2–7, and the preliminary conclusions are shown in section “Discussion.”

**TABLE 2:** The main effect of explosion experiment scene on escape behavior.

Behavior	Non-explosion c_1_	Explosion c_2_	Main effect c_2_−c_1_
Response time (s)	1.1223	0.4282	–0.6941
Speed (m/s)	0.9510	1.8070	0.8560
Direction	0.1383	–0.0376	–0.1759
Valance	0.4300	–1.3400	–1.7770
Arousal	1.6400	4.7700	3.1300

*P < 0.001.*

**TABLE 3:** The main effect of neuroticism on escape behavior.

Behavior	Low neuroticism c_1_	High neuroticism c_2_	Main effect c_2_−c_1_
Response time (s)	0.7871	0.7641	–0.0230
Speed (m/s)	1.3671	1.3903	0.0232
Direction	0.0258	–0.1959	–0.2217
Valance	–0.1598	–0.7330	–0.5732
Arousal	3.0361	3.3641	0.3280

*P < 0.001.*

**TABLE 4:** The main effect of extroversion on escape behavior.

Behavior	Low extroversion c_1_	High extroversion c_2_	Main effect c_2_−c_1_
Response time (s)	0.7989	0.7497	–0.0492
Speed (m/s)	1.3861	1.3713	–0.0148
Direction	0.0724	0.1046	0.0322
Valance	–0.5817	–0.3177	0.2640
Arousal	3.3990	2.9948	–0.4042

*P < 0.001.*

**TABLE 5:** The main effect of openness on escape behavior.

Behavior	Low openness c_1_	High openness c_2_	Main effect c_2_−c_1_
Response time (s)	0.7794	0.7704	–0.0090
Speed (m/s)	1.4013	1.3534	–0.0479
Direction	0.0938	0.0809	–0.0129
Valance	–0.5888	–0.3011	0.2877
Arousal	3.2664	3.1344	–0.1320

*P < 0.001.*

**TABLE 6:** The main effect of agreeableness on escape behavior.

Behavior	Low agreeableness c_1_	High agreeableness c_2_	Main effect c_2_−c_1_
Response time (s)	0.7632	0.7866	0.0234
Speed (m/s)	1.3795	1.3786	–0.0009
Direction	0.1296	0.0487	–0.0809
Valance	–0.5258	–0.3883	0.1375
Arousal	3.2268	3.1845	–0.0423

*P < 0.001.*

**TABLE 7:** The main effect of conscientiousness on escape behavior.

Behavior	Low conscientiousness c_1_	High conscientiousness c_2_	Main effect c_2_−c_1_
Response time (s)	0.7823	0.7683	–0.014
Speed (m/s)	1.3676	1.3902	0.0226
Direction	–0.0127	0.1872	0.1999
Valance	–0.5808	–0.3317	0.2491
Arousal	3.1818	3.2277	0.0459

*P < 0.001.*

The interaction effect model between personality traits and experimental explosion on escape behavior (T, v, D) and emotional impact (V, A) is shown in Tables 8–12.

**TABLE 8:** Interaction of neuroticism and experimental scene on individual emotional impact and escape behavior.

Behavior	Low neuroticism c_1_	High neuroticism c_2_	Explosion scene c_4_	High neuroticism explosion scene c_24_
Response time (s)	1.1206	1.1239	–0.6670	–0.0527
Speed (m/s)	0.9340	0.9671	0.8662	–0.0199
Direction	0.5155	0.5922	–0.0515	0.0224
Valance	0.8041	0.0777	–1.9278	0.3065
Arousal	1.6082	1.6699	2.8557	0.5327

*P < 0.001.*

**TABLE 9:** Interaction of extroversion and explosion scene on individual emotional impact and escape behavior.

Behavior	Low extroversion c_1_	High extroversion c_2_	Explosion scene c_4_	Low extroversion non-explosion scene c_13_
Response time (s)	1.0652	1.0691	–0.6389	0.1063
Speed (m/s)	0.9994	0.9574	0.8278	–0.0542
Direction	0.4904	0.5417	0.0000	0.0769
Valance	0.3806	0.5938	–1.8229	–0.1018
Arousal	1.9175	1.4583	3.0729	–0.1098

*P < 0.001.*

**TABLE 10:** The interaction of openness and explosion scene on individual emotional impact and escape behavior.

Behavior	Low openness c_1_	High openness c_2_	Explosion scene c_4_	Low openness non-explosion scene c_13_
Response time (s)	0.9950	1.0717	–0.6025	0.1713
Speed (m/s)	0.9964	0.9335	0.8399	–0.0301
Direction	0.4537	0.5161	0.0323	0.1351
Valance	0.4102	0.6237	–1.8495	–0.1485
Arousal	1.9187	1.6452	2.9785	–0.2832

*P < 0.001.*

**TABLE 11:** Interaction of agreeableness and experimental scene on individual emotional impact and escape behavior.

Behavior	Low agreeableness c_1_	High agreeableness c_2_	Non-explosion scene c_3_	Low agreeableness explosion scene c_14_
Response time (s)	0.3993	0.434	0.7052	0.0227
Speed (m/s)	1.8273	1.8131	−0.869	–0.0268
Direction	0.5185	0.4951	4.85E-02	0.0176
Valance	–1.9115	–1.4369	2.0971	0.6744
Arousal	4.7266	4.7282	−3.0874	0.0879

*P < 0.001.*

**TABLE 12:** Interaction of conscientiousness and experimental scene on individual emotional impact and escape behavior.

Behavior	Low conscientiousness c_1_	High conscientiousness c_2_	Non-explosion scene c_3_	Low conscientiousness non-explosion scene c_13_
Response time (s)	0.4121	0.4439	0.6488	0.0916
Speed (m/s)	1.7913	1.8224	−0.8644	0.0170
Direction	0.4747	0.5545	3.96E-02	0.0800
Valance	–1.4444	–1.2376	1.8119	–0.0846
Arousal	4.798	4.7426	−3.0297	–0.2026

*P < 0.001.*

## Discussion

According to the main effect model from Tables 2–7, the results shows that: in the influence of escape behavior (T,v,D) and emotional impact (V,A) mode, experimental explosion E, personality neuroticism N, extroversion Ex, openness O, agreeableness A, and conscientiousness C have significant main effect (*P* < 0.001), and the significant effect is shown in Table 13.

**TABLE 13:** The main effects of explosion experiment and personality dimensions on individual emotional impact and escape behavior.

Behavior	Explosion vs. non-explosion	High neuroticism vs. low neuroticism	High extroversion vs. low extroversion	High openness vs. low openness	High agreeableness vs. low agreeableness	High conscientiousness vs. low conscientiousness
Response time	−	−	−	−	+	−
Speed	+	+	−	−	−	+
Direction	−	−	+	−	−	+
Valance	−	−	+	+	+	+
Arousal	+	+	−	−	−	+

*P < 0.001.*

The main effect of explosion scene or high neuroticism on escape behavior and emotional impact is as follows: when it is negative to escape response, it is positive to escape speed, negative to escape direction, negative to emotional valence, and positive to emotional arousal. For the escape behavior mode, the results show that the subjects tend to shorten the escape response time, accelerate the escape speed, and reduce the probability of subjects choosing to escape in the explosion scene and high neuroticism. For the emotional impact of individuals, the results show that subjects tend to reduce the emotional valence score and enhance the emotional experience under explosive scenes or high neuroticism. In other words, participants tend to have a shorter response time, a faster escape rate, and a behavioral tendency to choose to escape near the door, all of which tend to obtain a relatively negative and strong emotional experience.

The main effect of high extroversion on escape behavior and emotional impact is: it has a negative effect on escape response, negative effect on escape speed, positive effect on escape direction, positive effect on emotional valence, and negative effect on emotional arousal. The results show that in the case of high extroversion, the participants tend to shorten the escape response time, slow down the escape speed, and increase the probability of the participants choosing the distant door to escape. They tend to increase the emotional valence score and reduce the emotional experience excitement. In other words, under high extraversion, participants tend to have a shorter response time, slower escape speed, a behavioral tendency to choose to escape near the door and to get relatively positive, and weak emotional experience.

The main effect of high openness and high agreeableness on individual escape behavior and emotional impact is: it has a negative effect on escape speed, negative effect on escape direction, positive effect on emotional valence, and negative effect on emotional arousal. For the escape behavior, the results show that under the condition of high openness and agreeableness, participants tend to slow down the escape speed and reduce the probability of participants choosing to escape from the distant door. They tend to increase the emotional valence score and reduce the emotional experience excitement. In other words, under the condition of high openness and agreeableness, subjects tend to have a slower escape speed, choose the behavior tendency of near-door escape, and tend to obtain relatively positive and weak emotional experience.

The main effect of high rigor on individual escape behavior and emotional impact is: it has a negative effect on escape response, positive effect on escape speed, positive effect on escape direction, positive effect on emotional valence, and positive effect on emotional arousal. The results show that under the condition of high conscientiousness, subjects tend to reduce the escape response, accelerate the escape speed, increase the probability of subjects choosing to escape from the distant door and tend to increase emotional valence score, and increase emotional experience excitement.

According to the interaction effect model from Tables 8–12, personality neuroticism N, extroversion Ex, openness O, agreeableness A, and conscientiousness C significantly interact with explosion experiment E on escape behavior (i.e., response time T, speed v, and direction D) and emotional impact (i.e., valence V and arousal A), and the significant effect is shown in Table 14.

**TABLE 14:** Interaction effects of personality dimensions and explosion experiments on individual emotional impact and escape behavior.

Behavior	High neuroticism explosion N_H_ E_Y_	Low extroversion non-explosion E_*x*_L__ E_N_	Low openness non-explosion O_L_ E_N_	Low explosion A_L_ E_Y_	Low conscientiousness explosion C_L_ E_N_
Response time	−	+	+	+	+
Speed	−	−	−	−	+
Direction	+	+	+	+	+
Valence	+	−	−	+	−
Arousal	+	−	−	+	−

*P < 0.001.*

The interaction effect of high neuroticism or low agreeableness of personality trait and explosive experimental scene is: it has a negative effect on escape speed, positive effect on escape direction, positive effect on emotional valence, and positive effect on emotional arousal. For the escape behavior pattern, the results show that the interaction term of high neuroticism, low agreeableness, and explosive scene tend to slow down the escape rate and increase the probability of participants choosing to escape from the distant door. In other words, subjects with high neuroticism and low agreeableness tend to have a slower escape speed and a tendency to choose to escape far away in an explosion scenario. For high neuroticism, low agreeableness subjects are subject to emotional impact; the results show that the high neuroticism, low agreeableness subjects, in the explosion scene, tend to increase the emotional valence, arousal score, that is, making subjects get a more positive, more intense, excited emotional experience.

The interaction effect of personality with low extroversion, low openness, low conscientiousness, and nonexplosion experimental scene is as follows: it has a positive effect on escape reaction, positive effect on escape direction, negative effect on emotional valence, and negative effect on emotional arousal. For the escape behavior pattern, the results show that low extroversion, low openness, low conscientiousness, and interaction with explosion scenes tend to increase the probability of participants choosing to escape from the distant door. In other words, participants with low extroversion, low openness, and low conscientiousness tend to escape from the distant door when they have a longer response time in the nonexplosion condition. For subjects with low extroversion, low openness, and low conscientiousness, the results show that they tend to reduce emotional valence and arousal scores in nonexplosion scenarios, that is, making subjects obtain neutral and less excited emotional experience.

In conclusion, by comparing the results of main effect and interaction effect, the following conclusions are obtained:

1. Participants with high neuroticism, in the explosion scenario, tend to have a slower escape speed, choose the distant door to escape, and get more positive, stronger, excited emotional experience.
2. Participants with low extroversion, low openness, and low conscientiousness tend to choose the distant door to escape when they have a longer response time in the nonexplosion scenario. They get a neutral, less excited emotional experience.
3. If only considering the explosion scene or high neuroticism, participants tend to react quickly, escape quickly, choose the near door to escape, and tend to get relatively negative, strong emotional experience.
4. If only considering the high extraversion, participants tend to react quickly, escape slowly, choose the distant door to escape, and tend to get relatively positive, weak emotional experience.
5. If only considering high openness and high agreeableness, participants tend to slow down, choose the near door to escape, and tend to get relatively positive, weak emotional experience.
6. If only considering high conscientiousness, participants tend to react quickly, escape quickly, choose the distant door to escape, and tend to get relatively positive, strong emotional experience.

To summarize, neuroticism refers to incapacity to maintain emotional stability, i.e., groups with high neuroticism are more emotionally susceptible and, therefore, feel more intense emotional experiences. The high neuroticism group shows susceptibility to high-intensity negative stimuli from the explosion, reacting faster and experiencing more intense negative emotions. Extraversion reflects optimistic, adventurous traits. And low extraversion groups have a more stable emotional experience. Groups with high openness and high agreeableness are imaginative and forthright and, therefore, slow to escape, with weak but positive emotional experience. Conscientiousness, meanwhile, is a trait of organization, caution, and restraint, so highly conscientiousness participants have fast reaction and escape times with relatively positive emotional experiences.

## Conclusion

In this study, the modeling method of the general linear model is used to design the simulation experiment of traffic accidents under different personal traits and explosion scenarios. The new problems of the influence of different personal traits and traffic accidents on individual escape are modeled, and the highly neurotic individuals are obtained. In the explosion scenario, they have the tendency to choose the door, which is far from them with a shorter response time and slower speed to escape. This will lay a foundation for revealing the individual escape law in traffic accidents and provide a theoretical model reference for the timely and effective escape of people.

## Data Availability Statement

The datasets presented in this article are not readily available because participants only gave ethical consent for this project, and not for further distribution outside the research team. Requests to access the datasets should be directed to c_tong@swu.edu.cn or the corresponding author WA.

## Ethics Statement

The studies involving human participants were reviewed and approved by the School of Electronic and Information Engineering, Southwest University. The patients/participants provided their written informed consent to participate in this study.

## Author Contributions

YX: modeling and writing. WA: supervising. XX: building database and experiments. All authors contributed to the article and approved the submitted version.

## Conflict of Interest

YX and WA were employed by the company China Merchants Chongqing Communications Technology Research and Design Institute Co., Ltd. The remaining authors declare that the research was conducted in the absence of any commercial or financial relationships that could be construed as a potential conflict of interest.

## Publisher’s Note

All claims expressed in this article are solely those of the authors and do not necessarily represent those of their affiliated organizations, or those of the publisher, the editors and the reviewers. Any product that may be evaluated in this article, or claim that may be made by its manufacturer, is not guaranteed or endorsed by the publisher.
